# Identification of arboviruses and certain rodent-borne viruses: reevaluation of the paradigm.

**DOI:** 10.3201/eid0704.010431

**Published:** 2001

**Authors:** American Committee on Arthropod-borne Viruses, Subcommittee on InterRelationships among Catalogued Arboviruses

## Abstract

Diagnostic and epidemiologic virology laboratories have in large part traded conventional techniques of virus detection and identification for more rapid, novel, and sensitive molecular methods. By doing so, useful phenotypic characteristics are not being determined. We feel that the impact of this shift in emphasis has impaired studies of the biology of viruses. This position paper is a plea to the scientific and administrative communities to reconsider the importance of such information. We also suggest a revised paradigm for virus isolation and characterization and provide a rationale for accumulating biologic (phenotypic) information.


### Identification of Arboviruses and Certain Rodent-Borne Viruses: Reevaluation of the Paradigm

Diagnostic and epidemiologic virology laboratories have in large part traded conventional techniques of virus detection and identification for more rapid, novel, and sensitive molecular methods. By doing so, useful phenotypic characteristics are not being determined. We feel that the impact of this shift in emphasis has impaired studies of the biology of viruses. This position paper is a plea to the scientific and administrative communities to reconsider the importance of such information. We also suggest a revised paradigm for virus isolation and characterization and provide a rationale for accumulating biologic (phenotypic) information.

### Historical Background

Until about 10 years ago, arthropod-borne viruses (arboviruses) were isolated and then identified by methods now referred to as "classical." That is, clinical or field-collected samples were processed by methods originally established by yellow fever researchers at the Rockefeller Foundation (1-3). As these procedures were shared and adopted by essentially all laboratories conducting arbovirus surveillance and research, they became the standard for arbovirus laboratories worldwide. These techniques were developed to facilitate specific identification of viruses isolated from hematophagous arthropods, vertebrate animals, and human clinical samples. The general scheme included a) isolation of the virus; b) production of a virus seed or stock; c) production of a sucrose-acetone extracted antigen (often inactivated so that it could be used safely for serodiagnostic procedures); d) preparation of an antibody (usually hyperimmune mouse ascitic fluids); e) registration of the virus; and f) deposition of the virus as a voucher specimen in a reference collection (3,4). The accumulation of such reagents by arbovirus laboratories allowed the establishment of reference centers that, with the support and encouragement of the World Health Organization (5,6) and various national governments, distribute useful reagents to regional and local laboratories. Local laboratories, in turn, were then able to conduct serodiagnostic tests for antibody to newly recognized arboviruses, using standardized reagents for virus identification procedures. As an intentional by-product, a network of collaborating centers was established and an international spirit of cooperation and camaraderie evolved, as exemplified by the American Committee on Arthropod-Borne Viruses (ACAV) and its various subcommittees that take responsibility for collating a catalog of the recognized arthropod-borne and rodent-associated viruses (7), evaluating their safety, storing voucher specimens, and determining their antigenic relationships. The resulting catalog, entitled The International Catalogue of Arboviruses and Certain Other Viruses of Vertebrates, has long been the "bible" of arbovirologists. However, with the availability of newer molecular techniques and the current emphasis on genomics, many viruses now are detected by molecular means only. Consequently, few newly discovered viruses are now being registered in the arbovirus catalog, although hundreds of genomic sequences of arboviruses, hantaviruses, arenaviruses, and filoviruses are entered annually in GenBank or other sequence databases. The latter data provide little or no phenotypic information, and, although the ACAV is attempting to provide accessible online biological information regarding arboviruses and other viruses, progress has been slow, in part because of lack of funding and perception of needs. The ultimate goal is to merge genotypic information, such as that deposited in sequence databases, with phenotypic and epidemiologic information, such as that published in the arbovirus catalog, and thereby provide a more accurate and complete picture of the biological characteristics of each virus.

In the heyday of arbovirology (ca. 1960-1975), arbovirus laboratories were fully functional in many parts of the world and both government and institutional support was high. The levels of training, reagent availability, virus discovery, epidemiologic assessments, and research activities were likewise high. As new techniques were developed, the name of the group studying arboviral antigenic relationships was changed from the Subcommittee on *Immunological Relationships* Among Catalogued Arboviruses to the Subcommittee on *InterRelationships* Among Catalogued Arboviruses (SIRACA), to reflect the introduction of molecular techniques as adjunct tools for virus identification and characterization. Few could have predicted the rapid advances to be made or the detail to which the arboviruses would be characterized.

### The Apparent Paradigm Shift

As newer techniques (monoclonal antibodies for specific virus identification, immunohistochemistry, RNA fingerprinting, nucleic acid hybridization, and, in particular, polymerase chain reaction and nucleic acid sequencing) were introduced, the earlier techniques were replaced as front-line diagnostic tests, although they remained adequate for most purposes. One reason for this trend was that nucleic acid sequencing and monoclonal antibody mapping of proteins could be used for remarkably rapid and detailed analyses of virus identities and structures by using reagents that had better production consistency and were easier to standardize between laboratories. However, the reliance on genomic sequencing for virus identification has resulted in an apparent quandary: whether to use molecular or other methods for virus identification. Molecular techniques provide information regarding genotypic characteristics. Serologic techniques (hemagglutination inhibition, complement fixation, immunofluorescence using polyclonal or monoclonal antibodies, enzyme-linked immunosorbent assay, neutralization, and vaccination challenge) provide information regarding phenotype. These serologic techniques provide insight into protection and cross-protection against virus infection, information that is of essential epidemiologic and public health significance.

### Information Gained, Opportunities Lost

In reality, there is no quandary. Genotypic and phenotypic data are complementary; the phenotype is simply the outward observable characteristic of a virus as determined by its genotype. The genomic sequence provides the foundation for phenotypic expression, but it is not yet possible to deduce completely the phenotype of a virus solely from its genomic sequence. Although some antigenic properties can be determined by using recombinant antigens, most phenotypic characteristics of a virus (its host range, pathogenicity, cell and tissue tropisms, replication characteristics, and elicitation of protective immunity) still must be determined directly. Because of this, SIRACA continues to support the use of phenotypic assays to identify and classify newly discovered viruses. It is not always necessary to derive genome sequence information for appropriate classification. In fact, for some arboviruses, e.g., those of the families Bunyaviridae and Rhabdoviridae, so little genomic sequence information exists that virus identification must rely on serologic techniques.

### Reasons To Accumulate Phenotypic Information

To accurately phenotype a newly discovered virus, infectious virus must be available. Only with an actual isolate is it possible to obtain normal antigenic and other biologic information for comparison with the classical virus databases that have been accrued over many decades. Without a virus isolate, direct cross-protective assays cannot be conducted, and therefore the interrelationships by neutralization of newly recognized arboviruses, hantaviruses, arenaviruses, and filoviruses cannot be determined. Cross-neutralization relationships have been the basis by which most of these viruses have been classified and differentiated (8-15).

Recently, sequencing of virus genomes has opened the fields of viral phylogenetics and molecular epidemiology, allowing comparisons not possible by the older, classical methods. It is now possible to determine rapidly and with some certainty the sources of viruses causing dengue fever (16), West Nile fever (17,18), Venezuelan equine encephalitis (19), hantavirus pulmonary syndrome (20), Ebola fever (21), and outbreaks caused by many other viruses (22). Still, procedures appear to have outpaced process in the study of emerging and reemerging virus diseases.

### New Technology Creates New Problems

Detection of viral nucleic acid is not equivalent to isolating a virus. Some hantaviruses have been detected, sequenced and placed in a taxon, and the proteins of some have been expressed without the viruses having been isolated (23). Newly recognized hantaviruses have been described solely on the basis of genomic sequencing, without the agent ever being isolated or the appropriate phenotyping reagents being produced (24-26). Without an isolate, the pathogenic potential, association with human infections and illnesses, and cross-protectivity are difficult to assess. One of the reasons for this development is that agencies funding virus research have opted to support mainly molecular and genetic studies. This funding decision has had a direct effect on the type of virus research carried out at universities, as well as direct and indirect effects on faculty recruitment and graduate education. Research involving the new genetic technologies is promoted as “cutting edge” and “mechanistic,” while more classical phenotypic studies are referred to somewhat disparagingly as “descriptive.” In truth, both types of research are largely descriptive; genome sequencing and phylogenetic studies of viruses are the molecular equivalents of classical (phenotypic) studies of antigenic properties and antigenic interrelationships. However, both types of research are essential to our understanding of the mechanisms of viral pathogenesis, disease expression, and protective immunity.

Another reason for the lack of phenotypic information about most newly discovered viral pathogens is the increased number of restraints and regulations on the importation, use, and exchange of infectious viruses. The result has been to severely restrict their study to a relatively few high-security laboratories. Inactivated RNA or DNA samples of such agents can be obtained without the need for permits, which favors the use of molecular or genetic methods for studying new viruses. The filoviruses are a case in point. These viruses are extremely hazardous and must be handled under strict Biosafety Level 4 containment. Little is known about their antigenic interrelationships, cross-protectivities, and biological characteristics. Because of the hazards posed by working with these viruses, this is likely to remain the case for the foreseeable future. In contrast, nucleotide sequence analyses of filoviruses provide information adequate for epidemiologic and diagnostic purposes, as well as for phylogenetic studies. Such analyses cannot provide antigenic information for group placement (classification) by neutralization tests or tell us much about pathogenesis or protection. However, in view of their hazardous nature, it would seem prudent for most laboratories to continue assaying filoviruses by molecular techniques, rather than to attempt direct virus isolation.

Despite the remarkable advances in sequencing and phylogenetic analysis, there still is little agreement on the standardization of sequencing approaches, which portions of the genomes of these agents are “best” for designing primers for amplification and diagnostic purposes, and which genome regions will provide the most useful sequence information for taxonomic purposes (for example, the gene coding for the expression of an immunodominant epitope). Uniformity is the sine qua non of such comparisons.

Other issues also impact biological characterization of viruses. For example, little funding is available for the study of animal viruses that are not known or suspected to be pathogens of humans, livestock, or wildlife. The current system of research support in the United States does not encourage the study of orphan viruses until they emerge as proven pathogens, a significant departure from the previous longstanding and productive policy. Likewise, funding agencies have little interest in supporting field studies designed to isolate and identify new viruses. Much lip service is given to the need for biological inventories of species diversity (genetic resources), but in the case of viruses, little funding is available for such studies. As noted, restrictions on the shipment and exchange of some infectious agents, because of biosafety and bioterrorism concerns, have inhibited biological studies with many viruses. Further discussions of these issues are beyond the scope of this paper.

### A Solution?

SIRACA continues to emphasize the need for new virus isolates for reference and antigenic studies and for reagent production, even when such isolates are difficult to retrieve. We emphasize that the sources of new arboviruses, hantaviruses, arenaviruses, and filoviruses are field materials, not laboratories. Without support for continued field studies and continued virus isolation, including long-term storage of representative virus isolates, our knowledge of viral ecology, evolution, and disease emergence will continue to suffer.

In summary, remarkable advances in molecular genetics have allowed rapid and precise identifications of viruses and of their genomes; however, such characterizations thus far can provide only limited information about the phenotype and disease potential of a virus. In addition to more support for studies of viral ecology, pathogenesis, and disease potential, there is a need for serologic reagents with which classical studies can be done. We suggest that infectious materials, in the form of seed virus, be submitted to reference repositories, such as those at the University of Texas Medical Branch, Galveston, Texas; the Division of Vector-Borne Infectious Diseases, Centers for Disease Control and Prevention, Fort Collins, Colorado; and the Institut Pasteur, Paris, France. These and other reference centers are supported by home institutions, government agencies, and other funding sources and serve as repositories, rather like museums without the dust.

We suggest that viruses, not simply their genomes, be registered with ACAV, the specialty group on which the International Committee for Taxonomy of Viruses mainly depends for classification of the arboviruses, hantaviruses, arenaviruses, and filoviruses. Financial and enthusiastic and knowledgeable administrative support is needed to continue the task of updating the arbovirus catalog and making it available electronically. As with disease diagnosis, it is the process, not the procedure, that is critical to success.

**American Committee on Arthropod-borne Viruses, Subcommittee on InterRelationships Among Catalogued Arboviruses[1]**

### References

1. Clarke DH, Casals J. Techniques for hemagglutination and hemagglutination-inhibition with arthropod-borne viruses. Am J Trop Med Hyg 1958;7:561-73.
2. Theiler M, Downs WG. The arthropod-borne viruses of vertebrates. An account of the Rockefeller Foundation virus program, 1951-1970. New Haven: Yale University Press; 1973.
3. Work TH. Isolation and identification of arthropod-borne viruses. In: Lennette EH, Schmidt NJ, editors. Diagnostic procedures for viral and rickettsial diseases, 3rd ed. New York: American Public Health Organization, 1964. p. 312-55.
4. Beaty BJ, Calisher CH, Shope RE. Arboviruses. In: Schmidt NJ and Emmons RW, eds. Diagnostic procedures for viral, rickettsial and chlamydial infections, 6th ed. Washington: American Public Health Association; 1989. p. 797-855.
5. Casals J. Procedures for identification of arthropod-borne viruses. Bull World Health Org 1961;24:723-34.
6. Report of a WHO Scientific Group. Arthropod-borne and rodent-borne viral diseases. Tech Report Series 179. Geneva: World Health Organization; 1985.
7. Karabatsos N. International catalogue of arboviruses including certain other viruses of vertebrates, 3rd ed. San Antonio: American Society of Tropical Medicine and Hygiene; 1985.
8. Calisher CH, Shope RE, Brandt W, Casals J, Karabatsos N, Murphy FA, et al. Recommended antigenic classification of registered arboviruses. I. Togaviridae, *Alphavirus*. Intervirology 1980;14:229-32.
9. Tesh RB, Travassos APA, Travassos JS. Antigenic relationship among rhabdoviruses infecting terrestrial vertebrates. J Gen Virol 1983;64:169-76.
10. Travassos APA, Tesh R, Pinheiro FP, Travassos da Rosa JFS, Peterson NE. Characterization of eight new phlebotomus fever serogroup arboviruses (Bunyaviridae: *Phlebovirus*) from the Amazon Region of Brazil. Am J Trop Med Hyg 1983;32:1164-71.
11. Calisher CH. Antigenic classification and taxonomy of flaviviruses (family Flaviviridae) emphasizing a universal system for the taxonomy of viruses causing tick-borne encephalitis. Acta Virol 1988;32:469-78.
12. Calisher CH, Karabatsos N. Arbovirus serogroups: definition and geographic distribution. In: Monath TP, editor. The arboviruses: epidemiology and ecology, vol. 1. Boca Raton, FL: CRC Press; 1988. p. 19-58.
13. Calisher CH. History, classification, and taxonomy of viruses in the family Bunyaviridae. In: Elliott RM, editor. The Bunyaviridae. New York: Plenum Press; 1996. p. 1-17.
14. Howard CR. Antigenic diversity among the arenaviruses. In: Salvato MS, editor. The arenaviridae. New York: Plenum Press; 1993. p. 37-49.
15. Chu Y-K, Rossi C, LeDuc JW, Lee HW, Schmaljohn CS, Dalrymple J. Serological relationships among viruses in the *Hantavirus* genus, family Bunyaviridae. Virology 1994;198:196-204.
16. Wang E, Ni H, Xu R, Barrett AD, Watowhich SJ, Gubler DJ, et al. Evolutionary relationships of endemic/epidemic and sylvatic dengue viruses. J Virol 2000;74:3227-34.
17. Lanciotti RS, Roehrig JT, Deubel V, Smith J, Parker M, Steele K, et al. Origin of the West Nile virus responsible for an outbreak of encephalitis in the northeastern United States. Science 1999;286:2333-7.
18. Jia X-Y, Briese T, Jordan I, Rambaut A, Chi HC, Mackenzie JS, et al. Genetic analysis of West Nile New York 1999 encephalitis virus. Lancet 1999;354:1971-2.
19. Powers AM, Oberste MS, Brault AC, Rico-Hesse R, Schmura SM, Smith JF, et al. Repeated emergence of epidemic/epizootic Venezuelan equine encephalitis from a single genotype of enzootic subtype ID virus. J Virol 1997;71:6697-705.
20. Nichol ST, Ksiazek TG, Rollin PE, Peters CJ. Genetic identification of a hantavirus associated with an outbreak of acute respiratory illness. Science 1993;262:914-7.
21. Sanchez A, Ksiazek TG, Rollin PE, Miranda MEG, Trappier SG, Khan AS, et al. Detection and molecular characterization of Ebola viruses causing disease in human and nonhuman primates. J Infect Dis 1999;179(suppl 1):S164-S169.
22. Powers AM, Brault AC, Tesh RB, Weaver SC. Reemergence of chikungunya and o'nyong nyong viruses: evidence for distinct geographic lineages and distant evolutionary relationships. J Gen Virol 2000;8:471-9.
23. Schmaljohn C, Hjelle B. Hantaviruses: a global problem. Emerg Infect Dis 1997;3:95-104.
24. Lewis S, Morzunov SP, Rowe JE, Enria D, Pini N, Calderon G, et al. Genetic diversity and epidemiology of hantaviruses in Argentina. J Infect Dis 1998;177:529-38.
25. Hjelle B, Chavez-Giles F, Torrez-Martinez N, Yates T, Sarisky J, Webb J, et al. Genetic identification of a novel hantavirus of the harvest mouse, *Reithrodontomys megalotis*. J Virol 1994;68:6751-4.
26. Vincent MJ, Quiroz E, Gracia F, Sanchez AJ, Ksiazek TG, Kitsutani PT, et al. Hantavirus pulmonary syndrome in Panama: identification of novel hantaviruses and their likely reservoirs. Virology 2000;277:14-9.

[1]Charles H. Calisher, Chair; Carol D. Blair, Michael D. Bowen, Jordi Casals, Michael A. Drebot, Eric A. Henchal, Nick Karabatsos, James W. LeDuc, Patricia M. Repik, John T. Roehrig, Connie S. Schmaljohn, Robert E. Shope, Robert B. Tesh, and Scott C. Weaver
